# Treatment of Poorly Responsive Gamma-Hydroxybutyrate Withdrawal With Baclofen: A Case Report

**DOI:** 10.7759/cureus.25728

**Published:** 2022-06-07

**Authors:** William Lai, Jesse T Raposa, Roop Parlapalli

**Affiliations:** 1 Internal Medicine, Geisinger Health Systems, Wilkes-Barre, USA; 2 Internal Medicine, Geisinger Medical Center, Wilkes-Barre, USA; 3 Internal Medicine, Geisinger Health System, Scranton, USA

**Keywords:** date rape drug, drugs of abuse, analgesics, substance abuse, gamma hydroxybutyrate, ghb

## Abstract

Gamma-hydroxybutyrate (GHB) is a sedative often abused for its euphoric and relaxant effects. This case report looks to discuss a case of GHB intoxication in a 57-year-old gentleman, which resulted in an 11-day hospitalization due to withdrawal effects of his GHB dependence. His hospitalization and care primarily followed usual supportive care treatments; however, a novel use of baclofen to further expedite patient sedation reversal was done. This case report looks to explore the management of this patient's GHB toxicity and eventual resolution of symptoms using baclofen.

## Introduction

Gamma-hydroxybutyrate (GHB) intoxication is difficult to diagnose in patients as it is not detected by routine urine drug testing. Thus, the treatment and care of patients are often guided by patient history, clinical suspicion, or unused medication available for testing. The symptoms of GHB intoxication are nonspecific and similar to those of other central nervous system (CNS) depressants. The symptoms often include insidious central nervous depression, sometimes leading to obtundation with abrupt reversal [1]. The onset of symptoms is said to be as short as 30 minutes and last as long as 8 hours [2]. However, for patients who have recurrent use, withdrawal symptoms are more concerning because of their longer duration, often reported to be variable from days to weeks [3]. Withdrawal symptoms can be categorized by severity, with mild symptoms including diaphoresis, tremors, delirium, confusion, restlessness, insomnia, tremors, nystagmus, tachycardia, and hypertension. Moderate to severe symptoms often include agitation, hallucinations, and psychosis. This makes caring for GHB withdrawal patients tricky as the protracted window of care for these patients with sometimes severe symptoms as compared to other substances of abuse makes finding suitable care difficult to find. Fortunately, it is rare to see patients with GHB withdrawal symptoms as it is usually prevalent in patients who abuse the drug multiple times a day for months, if not years [3]. However, the rarity of GHB withdrawal means reversal treatments are poorly studied and often require long-protracted lengths of stay that can tax the healthcare system. The following case presentation looks at a patient who has chronically used GHB recreationally for the last 30 years and was brought to the hospital by emergency medical services for altered mental status.

## Case presentation

A 57-year-old man was brought to the hospital via emergency medical services after being found "rolling on the ground, thrashing, confused and disoriented." The patient was altered but interactive enough to admit to the intentional use of gamma-hydroxybutyrate. On further history review from his family members who were present at the bedside, the family revealed the patient had a longstanding history of GHB abuse for at least 30 years. The family also reports that he binges on GHB and has had multiple episodes of withdrawal, including aggressive and intermittent sedation whenever he loses access to the substance. These withdrawal symptoms often require hospital care due to severe agitation and altered mental status. On evaluation, he was noted to be somnolent, moving spontaneously, intermittently responsive though answering questions inappropriately, and confused. The patient had routine lab work including a complete metabolic panel, troponin, thyroid-stimulating hormone, salicylate level, lactate, acetaminophen level, ethanol level, urine drug toxicology, ammonia level, and creatinine kinase. The significant results included hypokalemia of 2.8 mmol/L, mildly elevated troponin of 32 ng/L, and elevated creatinine kinase of 6608 U/L, with the remainder of the findings reported in Table 1. He also had computerized tomography (CT) imaging of his head, which showed no acute intracranial deficits.

**Table 1:** Admission laboratory tests including complete metabolic panel, complete blood count, ammonia level, urine drug screen, salicylate level, acetaminophen level, creatinine kinase, urine blood, and urine red blood cells.

Test	Value	Units	Normal range
Sodium	143	mmol/L	135–146
Potassium	2.8	mmol/L	3.5–5.1
Chloride	100	mmol/L	98–107
Bicarbonate	30	mmol/L	22–32
Blood urea nitrogen	5	mg/dL	6–20
Creatinine	0.7	mg/dL	0.6–1.2
Glucose	142	mg/dL	70–120
Calcium	9.4	mg/dL	8.4–10.2
Lactate	2	mmol/L	0.4–2.0
White blood cell count	11.3	K/µL	4–10.8
Hemoglobin	15.3	g/dL	14–16.8
Hematocrit	43.6	%	40.0–48.4
Platelet	295	K/µL	140–400
Acetaminophen level	<5.0	µg/mL	10.0–30.0
Salicylates level	<0.3	mg/dL	5.0–30.0
Amphetamine	Negative	n/a	Negative
Benzodiazepine	Negative	n/a	Negative
Cannabinoid	Negative	n/a	Negative
Cocaine metabolite	Negative	n/a	Negative
Hydrocodone	Negative	n/a	Negative
Hydromorphone	Negative	n/a	Negative
Methadone	Negative	n/a	Negative
Morphine	Negative	n/a	Negative
Codeine	Negative	n/a	Negative
Oxycodone	Negative	n/a	Negative
Ethanol	Negative	n/a	Negative
Ammonia	20	µmol/L	11.0–32.0
Creatinine kinase	6608	U/L	39–308
Urine blood	Positive	n/a	Negative
Urine RBC	Negative	n/a	Negative

Subsequently, the patient was admitted for toxic encephalopathy and rhabdomyolysis. He was started on intravenous fluids and the Clinical Institute Withdrawal Assessment Alcohol Scale (CIWA) protocol with lorazepam dosing as needed with holding parameters if there were any signs of sedation. Over the next 24 hours, his mentation declined with worsening confusion and non-purposeful movement, requiring restraints and later dexmedetomidine infusion to better control his agitation. His CIWA scores were reported to be elevated from 20 to 26, warranting lorazepam dosing. Despite multiple days of supportive care, his fluctuations in agitation and somnolence persisted, with periods of a Glasgow coma scale (GCS) as low as 10. Critical care medicine was consulted for his poor mental status and airway watch. However, they were unable to transfer the patient to the intensive care unit for airway watch due to a lack of bed availability. Addiction medicine and neurology were consulted as the patient was suspected to be in GHB withdrawal. Given his prolonged somnolence with borderline GCS, they recommended further workup of his encephalopathy. The workup included magnetic resonance imaging (MRI) of the head, ethyl glucuronide level, and a fentanyl screen. The MRI head was not able to be done initially due to his tremulousness and frequent movement, but ethyl glucuronide levels and fentanyl screening were negative, as seen in Table 2, making GHB withdrawal likely.

**Table 2:** Additional drug tests requested by addiction medicine and neurology.

Test	Value	Units	Normal range
Fentanyl	Negative	n/a	Negative
Ethyl glucuronide level	0	ng/mL	<500

The medical team, including neurology consultants, elected to stop the lorazepam dosing with the CIWA protocol and instead started scheduled oxazepam and clonidine to better address withdrawal symptoms in the setting of elevated transaminases and preferences for longer-acting benzodiazepines. Despite these changes, the patient continued to be erratic with symptoms of agitation interplayed with somnolence. Gabapentin was then added in an attempt to better control symptoms of agitation in the belief that these symptoms were due to GHB withdrawal. Again, the patient continued to fluctuate between aggressive agitated states and lethargy. Given his airway concerns and lack of critical care beds, addiction medicine and neurology recommended the addition of baclofen 10 milligrams orally three times daily to his GHB withdrawal treatment regimen. Within the first day, his agitation improved to the point that he was weaned off of his dexmedetomidine. Within two days, he had significant improvement in his delirium, agitation, and sedation, in which he was coherent enough to answer questions appropriately. He began to follow all commands and was fully oriented. Given his improving mental state, he was eventually found to be stable for discharge. The patient was continued on a baclofen, oxazepam, and clonidine taper and discharged home per the patient's request after education on the importance of rehabilitation and monitoring. 

## Discussion

We have discussed a case of a patient with gamma-hydroxybutyrate withdrawal symptoms that was successfully treated with baclofen. This case did not seem to have any confounding factors as his alcohol, drug screen, and specific toxicology came back negative but cannot fully be refuted as the patient's history of the events leading to his altered state was not clear. It seems that his symptoms of confusion were primarily driven by his GHB withdrawal. To understand why baclofen was chosen, we will discuss the physiology and history of GHB. GHB is an organic metabolite of gamma-aminobutyric acid (GABA). Synthetic forms of the neurotransmitter are considered dissociative anesthetics and have been used as a drug of abuse for their sedative, amnesic, and euphoric effects. First used in the 1980s, it was FDA approved for the treatment of narcolepsy as well as for its analgesic effects as a schedule III medication. However, its use was found to be of no clinical benefit, so it fell out of favor. However, this medication found its way to recreational use starting in the early 1990s for the aforementioned sedative, euphoric, aphrodisiac, disinhibitory, and amnesiac effects it provides while being undetectable on routine drug screenings [4]. It has been implicated in both self-abuse and sexual assault cases due to its sudden effect and amnesic properties [5]. GHB has also found common use in the weight lifting community for its unsubstantiated reports of growth hormone, sleep, and fat metabolism benefits [6]. Typically, GHB is taken orally as a liquid or powder, with doses typically around one to three teaspoons. Its recreational use peaked in the 2000s, with as many as 5000 cases a year being presented to emergency rooms in the United States [7]. Today, toxicity effects from this medication are a rare occurrence, accounting for only 2400 cases out of 2.5 million total emergency room presentations for drug abuse in the United States [5]. However, with the advent of cryptocurrencies and nonregulated drug procurement sites such as Silk Roads on the dark web, the acquisition and abuse of this medication have become far easier than ever before [8]. The effects of the medication are reported to begin within 15 minutes of ingestion and last two to four hours [9].

GHB is not a commonly used substance; however, what is known is that it and its precursors, gamma-butyrolactone (GBL) and 1,4-butanediol (1,4-BD), act on GABA-B receptors. GABA receptors have an inhibitory role in the nervous system and, thus, GABA-B agonism results in CNS depression. Some cases of GHB withdrawal can be effectively managed with long-acting benzodiazepines. If these medications do achieve control of withdrawal symptoms, then they can be tapered as symptoms are controlled. Patients that are poorly responsive to benzodiazepines can be trialed barbiturates which also act on GABA-A receptors. Mood stabilizers such as gabapentin and antipsychotics have also been considered for GHB withdrawal as their mechanisms of action do not directly act on GABA receptors but do have similar inhibitory effects. In this case, the patient was trialed on oxazepam, a hepatically safe, long-acting benzodiazepine, without improvement. Gabapentin and clonidine were then added for symptom control, both of which again did not help.

Baclofen was then considered for this patient as a temporizing measure. Baclofen acts on GABA-B receptors and theoretically can be used in the reversal of GHB withdrawal symptoms. GABA-B receptor agonism via baclofen use has been reported in case studies and has shown better efficacy compared to the standard use of benzodiazepines, showing that this is not a standalone finding [10]. However, this is not well studied in large randomized controlled trials given the rarity of the disorder. There have been animal studies done to suggest that there is good standing for baclofen use as compared to benzodiazepines, which showed benefits for GABA-B agonism versus GABA-A agonism [11]. Since benzodiazepines specifically act via GABA-A receptors, this may have limited our ability to abate the GHB-related withdrawal symptoms. Baclofen's targeted GABA-B activity proved efficacious in reversing our patient's withdrawal symptoms of somnolence and aggression. Baclofen's use in these scenarios has been recommended by some pharmacological societies [12]. This, however, has not been adopted into practice by all and is often used only under the advisement of addiction medicine or neurology.

## Conclusions

As we enter a more digital age with better access to substances like GHB, it becomes all the more important to be vigilant in our history taking and suspicions of drug abuse, as in this patient. With more than the average length of stay expected at around four to six weeks for GHB toxicity compared to alcohol intoxication, using Baclofen in suspicious cases of GHB-related intoxication and withdrawal seems to be a promising option. Its use, in this case, was shown to reduce the duration of symptoms and concomitant side effects from substance abuse during the hospital stay, including pneumonia, urinary tract infections, and hospital delirium. Further randomized control trials to better study this therapy as compared to supportive management will be needed to better elucidate the efficacy of this promising solution to GHB withdrawal.

## References

[1] Van Sassenbroeck DK, De Neve N, De Paepe P, Belpaire FM, Verstraete AG, Calle PA, Buylaert WA (2007). Abrupt awakening phenomenon associated with gamma-hydroxybutyrate use: a case series. Clin Toxicol (Phila).

[2] Schep LJ, Knudsen K, Slaughter RJ, Vale JA, Mégarbane B (2012). The clinical toxicology of γ-hydroxybutyrate, γ-butyrolactone and 1,4-butanediol. Clin Toxicol (Phila).

[3] Kamal RM, van Noorden MS, Wannet W, Beurmanjer H, Dijkstra BA, Schellekens A (2017). Pharmacological Treatment in γ-Hydroxybutyrate (GHB) and γ-Butyrolactone (GBL) Dependence: Detoxification and Relapse Prevention. CNS Drugs.

[4] Dyer JE (1991). Gamma-Hydroxybutyrate: a health-food product producing coma and seizure like activity. Am J Emerg Med.

[5] Bosman IJ, Verschraagen M, Lusthof KJ (2011). Toxicological findings in cases of sexual assault in the Netherlands. J Forensic Sci.

[6] Giorgetti A, Busardò FP, Giorgetti R (2022). Toxicological characterization of GHB as a performance-enhancing drug. Front Psychiatry.

[7] Drug Abuse Warning Network (DAWN), Drug Abuse Warning Network, 2011 2011 (2022). National Estimates of Drug Related Emergency Department Visits. U.S. Department of Health and Human Services, Substance Abuse and Mental Health Administration, Rockville, MD. https://www.samhsa.gov/data/report/national-estimates-drug-related-emergency-department-visits-2004-2011-all-visits.

[8] (2022). European Monitoring Centre for Drugs and Drug Addiction (EMCDDA). EU Drug Markets Report 2019. Luxembourg.

[9] Ries R, Fiellin D, Miller S, Saitz R: 4th Edition (2009). Principles of Addiction Medicine.

[10] Habibian S, Ahamad K, McLean M, Socias ME (2019). Successful management of gamma-hydroxybutyrate (GHB) withdrawal using baclofen as a standalone therapy: a case report. J Addict Med.

[11] Smith MA, Gergans SR, Lyle MA (2006). The motor-impairing effects of GABA(A) and GABA(B) agonists in gamma-hydroxybutyrate (GHB)-treated rats: cross-tolerance to baclofen but not flunitrazepam. Eur J Pharmacol.

[12] Lingford-Hughes AR, Welch S, Peters L, Nutt DJ (2012). BAP updated guidelines: evidence-based guidelines for the pharmacological management of substance abuse, harmful use, addiction and comorbidity: recommendations from BAP. J Psychopharmacol.

